# Evaluation of Tumor Viability for Primary and Bone Metastases in Metastatic Castration-Resistant Prostate Cancer Using Whole-Body Magnetic Resonance Imaging

**DOI:** 10.1155/2018/4074378

**Published:** 2018-06-07

**Authors:** Hiromichi Iwamura, Yasuhiro Kaiho, Jun Ito, Go Anan, Nozomi Satani, Tomonori Matsuura, Ryo Tamura, Kazuhiro Murakami, Kaneki Koyama, Makoto Sato

**Affiliations:** ^1^Department of Urology, Tohoku Medical and Pharmaceutical University, 1-12-1 Fukumuro, Miyaginoku, 983-8512 Sendai, Japan; ^2^Department of Radiology, Tohoku Medical and Pharmaceutical University, 1-12-1 Fukumuro, Miyaginoku, 983-8512 Sendai, Japan; ^3^Department of Pathology, Tohoku Medical and Pharmaceutical University, 1-12-1 Fukumuro, Miyaginoku, 983-8512 Sendai, Japan

## Abstract

In contrast to bone scan and computed tomography (CT), which depend on osteoblastic response to detect bone metastasis, whole-body magnetic resonance imaging (WB-MRI) may be able to directly detect viable tumors. A 75-year-old male who had progressive metastatic prostate cancer during primary androgen deprivation therapy was referred to our hospital. Although bone scan and CT showed multiple bone metastases, WB-MRI suggested nonviable bone metastasis and viable tumor of the primary lesion. Prostate needle biopsy demonstrated viable prostate cancer cells from 10 of 12 cores. In contrast, CT-guided needle biopsy from bone metastasis of the lumbar vertebra revealed no malignant cells. Based on these findings, we reasoned that viable tumor cells inducing disease progression may primarily exist in the primary lesions and not in the metastatic lesions, and combined prostate radiotherapy and systemic hormonal therapy resulted in successful clinical response and disease control. The use of WB-MRI to detect viable disease lesions may enable us to design optimal treatment strategies for patients with metastatic castration-resistant prostate cancer.

## 1. Introduction

As the disease condition of metastatic prostate cancer may change over time, it is necessary to evaluate and adjust its corresponding treatment as necessary, according to the given circumstances. Although bone scan and computed tomography (CT) have been the most widely used methods of evaluating metastases of prostate cancer, accurately evaluating bone metastases is often difficult because bone scan and CT depend on the osteoblastic response induced by tumor cells infiltrating the bone. In addition, bone scan and CT cannot evaluate the prostate. In contrast, whole-body magnetic resonance imaging (WB-MRI) allows direct detection of viable tumors not only in the metastatic sites but also in the primary lesion, owing to the method's excellent soft-tissue contrast, high spatial resolution, and lack of ionizing radiation [1]. Although several meta-analyses have shown that WB-MRI has greater diagnostic accuracy than bone scan and CT when detecting primary and metastatic lesions in patients with prostate cancer [2, 3], there is little evidence on whether the lesions diagnosed by WB-MRI truly contain pathologically viable tumor cells in metastatic castration-resistant prostate cancer (CRPC). Herein, we present an informative case of metastatic CRPC wherein the consistency of our WB-MRI and histopathological findings aided our decision in combining prostate radiotherapy (RT) and systemic hormonal therapy.

## 2. Case Presentation

**Figure 1:**
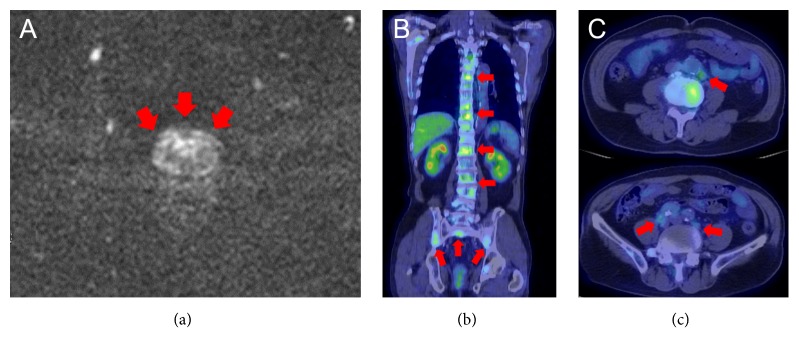
Image findings at initial diagnosis. Pelvic MRI (DWI) (a) showed extensive high signal of the prostate. FDG-PET/CT revealed multiple spine and pelvic bone (b) and para-aortic and pelvic lymph node metastases (c). MRI, magnetic resonance imaging; DWI, diffusion-weighted imaging; FDG-PET/CT, fluorodeoxyglucose-positron emission tomography/computed tomography.

A 75-year-old male visited a urological practitioner because of nocturia. An elevated serum prostate specific antigen (PSA) level of 76.2 ng/mL was observed, and digital rectal examination showed diffuse induration of the prostate. Pelvic MRI demonstrated extensive high signal of the prostate in diffusion-weighted imaging (DWI) (Figure 1(a)). Fluorodeoxyglucose-positron emission tomography/CT revealed multiple spine and pelvic bone and para-aortic and pelvic lymph node metastases (Figures 1(b) and 1(c)). Based on these findings, the patient was diagnosed with metastatic prostate cancer (cT3aN1M1b) and treated without prostate needle biopsy, with primary androgen deprivation therapy (ADT), including a GnRH antagonist (degarelix) and anti-androgen agent (bicalutamide). The PSA level immediately declined and reached nadir (0.23 ng/mL) after 8 months. However, 15 months after the start of ADT (PSA level, 2.33 ng/mL), the patient was diagnosed with CRPC and referred to our hospital.

**Figure 2:**
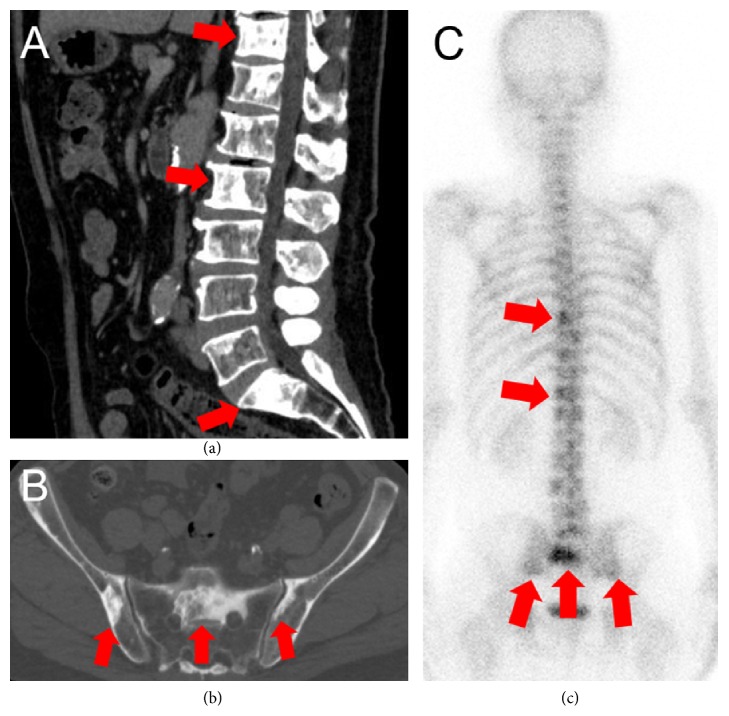
Imaging findings at the time of CRPC diagnosis. CT showed multiple osteoblastic lesions in the spine (a) and pelvic bone (b). Bone scan confirmed multiple accumulations at the same bone sites as CT (c). CRPC, castration-resistant prostate cancer; CT, computed tomography.

**Figure 3:**
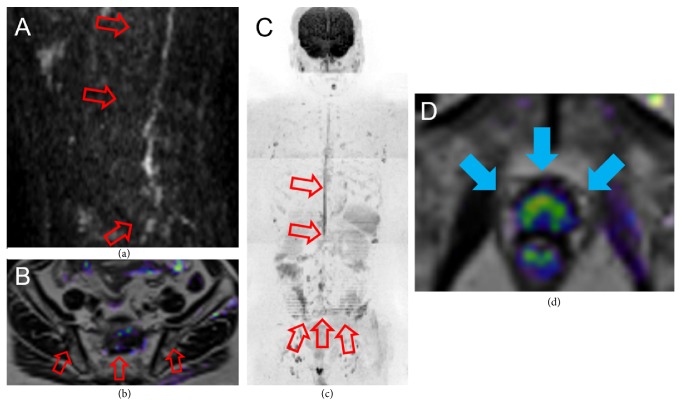
WB-MRI at the time of CRPC diagnosis. Sagittal DWI of the spine (a), coronal DWI + T2 fusion (b), and whole-body DWI (c) showed few high signals. Coronal DWI + T2 fusion of the pelvis (d) revealed diffuse high signal in the prostate. WB-MRI, whole-body magnetic resonance imaging; CRPC, castration-resistant prostate cancer; DWI, diffusion-weighted imaging.

To evaluate the patient's current disease status, we performed CT, bone scan, and WB-MRI. We observed discrepancies between the WB-MRI, bone scan, and CT. CT showed multiple osteoblastic lesions in the spine and pelvic bone (Figures 2(a) and 2(b)) and shrunken para-aortic lymph nodes. Bone scan similarly showed multiple accumulations at the same bone sites as the CT (Figure 2(c)). However, these osteoblastic lesions showed almost no high signal in DWI of WB-MRI, suggesting that the lesions did not have viable tumor cells (Figures 3(a)–3(c)). In contrast, the primary lesion had a diffuse high signal remaining in DWI of WB-MRI (Figure 3(d)).

**Figure 4:**
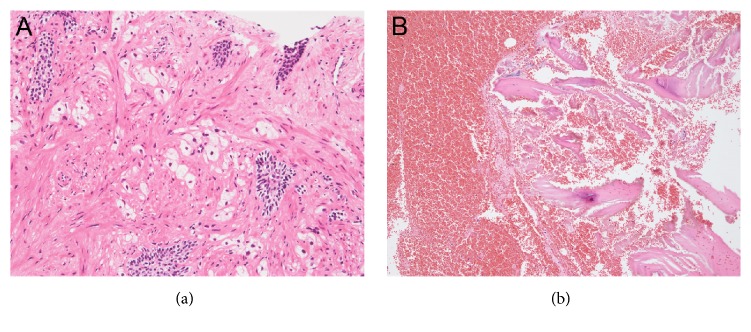
Histopathological findings at the time of CRPC diagnosis. Prostate needle biopsy detected viable prostate cancer cells from 10 of 12 cores (a). Bone needle biopsy from the second lumbar vertebra demonstrated no malignant cells (b). CRPC, castration-resistant prostate cancer.

**Figure 5:**
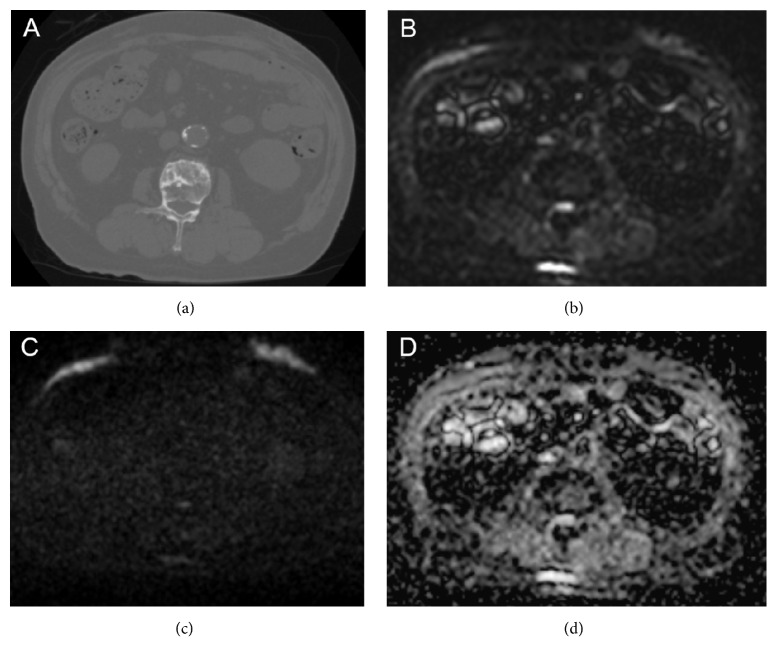
Imaging findings of MRI and CT in the second lumbar vertebra where CT-guided needle biopsy was performed. (a) CT revealed an osteoclastic lesion in the second vertebral body (885.4 HU). *b* = 0 (b) and *b* = 1200 (c) of DWI displayed no high signal intensity in the second lumbar vertebra. (d) The ADC map exhibited low signal intensity in the second lumbar vertebra (the ADC value was 0.498 × 10^−3 ^mm^2^/s). MRI, magnetic resonance imaging; CT, computed tomography; HU, Hounsfield unit; DWI, diffusion-weighted imaging; ADC, apparent diffusion coefficient.

We then performed histopathological examinations of both the prostate and the vertebra. The prostate needle biopsy demonstrated that 10 of 12 cores had viable prostate cancer cells (Figure 4(a)). Meanwhile, we performed the CT-guided needle biopsy from the osteoblastic lesion of the second lumbar vertebra, which was diagnosed with bone metastasis by CT and bone scintigraphy, to exclude vertebral bone metastasis of prostate cancer by an orthopedic unit of another hospital, which the patient visited owing to lumbago and bilateral lower limb paralysis. Histopathological examination of the osteoblastic lesion demonstrated no malignant cells (Figure 4(b)). Considering the results of the bone needle biopsy, we comprehensively reviewed the patient's image findings. A CT scan in the second lumbar vertebra revealed an osteoblastic change (885.4 Hounsfield unit), T1- and T2-weighted imaging of MRI revealed low signal intensity, and the ADC value was low (0.498 × 10^−3 ^mm^2^/s); however, *b* = 0 and *b* = 1200 of DWI exhibited no high signal intensity, suggesting a benign osteoblastic change induced by the treatment effect (Figures 5(a)–5(d)). With orthopedic conservative therapy in accordance with the diagnosis of lumbar hernia, the lumbago and bilateral lower limb paralysis completely resolved.

Based on the findings of our WB-MRI and histopathological examinations, we reasoned that viable tumor cells inducing disease progression may primarily exist in the primary lesions and not in the metastatic lesions. Then, to control the overall disease, we changed the patient's medication from bicalutamide to enzalutamide for potentially existing micrometastases and added prostate RT (74 Gy). After this, his elevated PSA immediately declined and was controlled at a level of <0.2 ng/mL.

## 3. Discussion

The present case has demonstrated the diagnostic accuracy of WB-MRI in a patient with metastatic CRPC, whose histopathological examination results were consistent with the WB-MRI results but not with the bone scan and CT results. The results of WB-MRI and histopathological examinations enabled us to provide optimal treatment to the patient, including combining prostate RT and systemic hormonal therapy.

Several meta-analyses have shown that WB-MRI has greater diagnostic performance for bone metastasis than bone scan and CT. The sensitivity and specificity of WB-MRI in the detection of bone metastasis were found to be ≥95%, whereas the equivalent measures for bone scan were just 78% and 85%, respectively, and for CT, just 77% and 83%, respectively [2–4]. In addition, bone scan and CT can show false-negative results for early and small bone metastasis, which are insufficient to produce a detectable osteoblastic response, and false-positive results for benign osteoblastic lesions. Similar to WB-MRI, novel imaging modalities, including ^18^F-NaF and ^18^F-fluorocholine PET/CT, demonstrated a higher diagnostic accuracy of bone metastasis in the past 10 years [5]. The diagnostic sensitivity and specificity of ^18^F-NaF PET/CT for bone metastasis were both 100% and those of ^18^F-fluorocholine PET/CT were 91% and 99%, respectively. Similarly, Beheshti et al. [6] reported that bone metastasis could be categorized into three groups based on the findings of ^18^F-fluorocholine PET/CT as follows: (a) bone marrow involvement (positive on ^18^F-fluorocholine PET and negative on CT); (b) typically osteoblastic but less often osteoclastic lesions (positive on ^18^F-fluorocholine PET and CT); and (c) densely sclerotic lesions (negative on ^18^F-fluorocholine PET and positive on CT). The densely sclerotic lesions that exhibited no metabolic uptake of ^18^F-fluorocholine could be attributable to the therapy-induced apoptosis of cancerous cells. More recently, ^68^Ga-prostate-specific membrane antigen (PSMA) PET/CT has been reported as a promising imaging modality, particularly for the detection of biochemical recurrence. Despite the cut-off value of PSA failure being <1.0 ng/mL, the detection rate of recurrence sites in patients with PSA failure following radical prostatectomy was 50% [7]. Tulsyan et al. [8] compared ^68^Ga-PSMA PET/CT with WB-MRI for the staging of 36 high-risk patients with prostate cancer. ^68^Ga-PSMA-PET/CT detected a higher number of patients with regional and distant lymph nodes (29 and 5, respectively) compared with that with WB-MRI (20 and 5, respectively). In contrast, the findings of a primary lesion in ^68^Ga-PSMA-PET/CT corroborated those in WB-MRI only by 52.7%, implying that WB-MRI is better than ^68^Ga-PSMA-PET/CT in terms of the simultaneous evaluation of prostate and metastatic lesions. These new imaging modalities, including WB-MRI, could contribute to offer precision medicine to patients with metastatic prostate cancer.

It has been reported that WB-MRI can directly detect tumor viability, with the support of DWI [9]. To evaluate treatment response in cases of bone metastases, decreases in volume and increases in median apparent diffusion coefficient (ADC) value are calculated by WB-MRI. Perez-Lopez et al. [10] recently reported that decreased tumor volume and increased ADC were correlated with PSA improvements in patients with CRPC by treatment using the PARP inhibitor olaparib. In contrast, CT and bone scan often show false-positives for the “flare phenomenon” (increased osteoblastic activity occurring in response to treatment) and posttreatment scarring [11]. In addition to evaluating metastatic lesions, WB-MRI can simultaneously evaluate the treatment response of the prostate. Recent studies have demonstrated the usefulness of DWI for evaluating therapeutic changes of the prostate [12, 13]. For example, Kim et al. reported that the mean ADC values of tumors in the prostate significantly increased after ADT compared with pretreatment values, whereas those of the benign tissues in the prostate significantly decreased [12]. Similarly, Wu et al. reported that the ADC values of tumors in the prostate significantly increased 3 months after RT, with an additional increase in ADC value between 3 and 12 months post-RT, whereas the ADC values of healthy control tissue in the same patients did not significantly change after RT [13]. These results indicate that DWI can differentiate viable from nonviable disease lesions. In our case, tumor viability was observed only in the prostate at the time of the CRPC diagnosis; thus, future tumor progression in the primary and/or metastatic lesions will be evaluated using changes in ADC values in DWI of WB-MRI.

It is noteworthy that diagnosis using WB-MRI for both primary and bone metastases in a patient with metastatic CRPC was histopathologically proven. Regarding the consistency of MRI and pathological findings, Perez-Lopez et al. [14] investigated whether the MRI findings of bone metastasis in patients with CRPC correlated with pathological findings. They accordingly evaluated 43 bone marrow biopsies from 33 patients with metastatic CRPC, multiparametric MRI, and documented bone metastases. The median ADC value was significantly lower and median DWI signal was considerably higher in biopsies with tumor cells compared with those of nondetectable tumor cells. They deduced that changes in the DWI parameters and histological features could parallel the tumor progression and regression, perhaps, because of the response to treatment. Although it has not yet been established whether WB-MRI can always reflect heterogeneous viability of the prostate and metastatic lesions in patients with metastatic CRPC, the present case is valuable and informative, as the results of the histopathological examinations were consistent with those of WB-MRI and not with the bone scan or CT and resulted in changes being made to the patient's disease management. To our knowledge, this is the first report on a case where the diagnostic accuracy of WB-MRI for both the prostate and bone metastases in metastatic CRPC was substantiated with histopathological examinations.

Recently, local therapy for metastatic prostate cancer has attracted attention because the median overall survival rate of metastatic prostate cancer is still only 42 months, despite the availability of several new drugs such as docetaxel, enzalutamide, abiraterone, cabazitaxel, and radium-223 [15]. Recently, two large retrospective studies [16, 17] have shown the significant survival benefit of local therapy in addition to systemic therapy for patients with metastatic prostate cancer. Rusthoven et al. [16] examined 6,382 males with newly diagnosed metastatic prostate cancer, and prostate RT with ADT showed a superior median overall survival rate (55 versus 37 months) and 5-year overall survival rate (49% versus 33%) compared with ADT alone. Löppenberg et al. [17] investigated the oncological benefit of local therapy including prostate RT, radical prostatectomy, and brachytherapy in 15,501 patients with metastatic prostate cancer, and the 3-year overall survival rate was higher in the local therapy group than in the non-local treatment group (69% versus 54%). However, at present, no guidelines recommend local therapy for patients with metastatic prostate cancer because of the lack of prospective studies.

There are two hypotheses as to why local therapy for metastatic prostate cancer can improve oncological outcomes. The first hypothesis is the “abscopal effect” of RT. This is a phenomenon where local irradiation causes the shrinking not only of the irradiated tumor but also tumors outside the irradiation field [18]. This may be caused by a tumor-specific immune response elicited against the tumor antigens released from the tumor cells damaged by irradiation. The second hypothesis is the elimination of tumor “self-seeding” by circulating tumor cells (CTCs). CTCs from the primary tumor shed into the vasculature or metastasize into a secondary tissue and then return to the primary tumor and accelerate their growth [19]. Considering these hypotheses, patients who have viable tumor cells in the prostate may benefit from local therapy, even in CRPC, as was the case with our patient. Therefore, novel imaging tools such as WB-MRI that evaluate the viability of both primary and metastatic lesions under systemic therapy are useful to identify patients with metastatic CRPC who would benefit from local therapy.

This case report has several limitations. First, we do not have access to pretreatment prostate biopsy, baseline WB-MRI, and bone scan results because the patient's initial diagnosis and treatment occurred before his referral to our hospital. Second, we could not exclude the possibility of a false-negative result in the bone biopsy, although we performed the CT-guided bone needle biopsy via the osteoblastic lesion, which was diagnosed with bone metastasis by CT and bone scintigraphy, with an orthopedically appropriate technique to detect bone metastasis [20]. Furthermore, the decrease in PSA level was not completely attributable to the local therapy because enzalutamide was simultaneously administered for potentially existing micrometastases. Despite these limitations, the knowledge gained from the present case supports the use of WB-MRI to progress treatment strategies in cases of patients with metastatic CRPC.

Accurate evaluation of the viability of both primary and metastatic lesions in patients with metastatic CRPC using WB-MRI may enable us to select patients who would benefit from local therapy. Prospective studies are needed to validate the treatment strategy associated with the use of WB-MRI for metastatic CRPC.

## Supplementary Materials

Supplementary Materials The course of treatment of the patient is demonstrated. His elevated PSA immediately declined and was controlled at a level of <0.2 ng/mL after the prostate RT and the administration of enzalutamide.

## References

[1] (2012). Erratum: Wu LM, Gu HY, Zheng J, et al. Diagnostic value of whole-body magnetic resonance imaging for bone metastases: A systematic review and meta-analysis. J Magn Reson Imaging 2011;34:128-135. *Journal of Magnetic Resonance Imaging*.

[2] Shen G., Deng H., Hu S., Jia Z. (2014). Comparison of choline-PET/CT, MRI, SPECT, and bone scintigraphy in the diagnosis of bone metastases in patients with prostate cancer: a meta-analysis. *Skeletal Radiology*.

[3] Liu L., Cui L., Zhang X. (2015). Diagnostic Performance of Diffusion-weighted Magnetic Resonance Imaging in Bone Malignancy. *Medicine*.

[4] Yang H.-L., Liu T., Wang X.-M., Xu Y., Deng S.-M. (2011). Diagnosis of bone metastases: a meta-analysis comparing ^18^FDG PET, CT, MRI and bone scintigraphy. *European Radiology*.

[5] Beheshti M., Rezaee A., Geinitz H., Loidl W., Pirich C., Langsteger W. (2016). Evaluation of prostate cancer bone metastases with 18F-NaF and 18F-Fluorocholine PET/CT. *Journal of Nuclear Medicine*.

[6] Beheshti M., Vali R., Waldenberger P. (2010). The use of F-18 choline PET in the assessment of bone metastases in prostate cancer: Correlation with morphological changes on CT. *Molecular Imaging and Biology*.

[7] von Eyben F. E., Picchio M., von Eyben R., Rhee H., Bauman G. (2016). 68Ga-Labeled Prostate-specific Membrane Antigen Ligand Positron Emission Tomography/Computed Tomography for Prostate Cancer: A Systematic Review and Meta-analysis. *European Urology Focus*.

[8] Tulsyan S., Das C. J., Tripathi M., Seth A., Kumar R., Bal C. (2017). Comparison of 68 Ga-PSMA PET/CT and multiparametric MRI for staging of high-risk prostate cancer 68 Ga-PSMA PET and MRI in prostate cancer. *Nuclear Medicine Communications*.

[9] Reischauer C., Froehlich J. M., Koh D.-M. (2010). Bone metastases from prostate cancer: Assessing treatment response by using diffusion-weighted imaging and functional diffusion maps - Initial observations. *Radiology*.

[10] Perez-Lopez R., Mateo J., Mossop H. (2017). Diffusion-weighted imaging as a treatment response biomarker for evaluating bone metastases in prostate cancer: A pilot study. *Radiology*.

[11] Cook G. J. R., Azad G., Padhani A. R. (2016). Bone imaging in prostate cancer: the evolving roles of nuclear medicine and radiology. *Clinical and Translational Imaging*.

[12] Kim A. Y., Kim C. K., Park S. Y., Park B. K. (2014). Diffusion-weighted imaging to evaluate for changes from androgen deprivation therapy in prostate cancer. *American Journal of Roentgenology*.

[13] Wu X., Reinikainen P., Kapanen M., Vierikko T., Ryymin P., Kellokumpu-Lehtinen P.-L. (2017). Diffusion-weighted MRI provides a useful biomarker for evaluation of radiotherapy efficacy in patients with prostate cancer. *Anticancer Reseach*.

[14] Perez-Lopez R., Nava Rodrigues D., Figueiredo I. (2017). Multiparametric Magnetic Resonance Imaging of Prostate Cancer Bone Disease. *Investigative Radiology*.

[15] James N. D., Spears M. R., Clarke N. W. (2015). Survival with newly diagnosed metastatic prostate cancer in the docetaxel era: Data from 917 patients in the control arm of the STAMPEDE Trial (MRC PR08, CRUK/06/019). *European Urology*.

[16] Rusthoven C. G., Jones B. L., Flaig T. W. (2016). Improved survival with prostate radiation in addition to androgen deprivation therapy for men with newly diagnosed metastatic prostate cancer. *Journal of Clinical Oncology*.

[17] Löppenberg B., Dalela D., Karabon P. (2017). The Impact of Local Treatment on Overall Survival in Patients with Metastatic Prostate Cancer on Diagnosis: A National Cancer Data Base Analysis. *European Urology*.

[18] Mole R. H. (1953). Whole body irradiation; radiobiology or medicine?. *British Journal of Radiology*.

[19] Scott J. G., Basanta D., Anderson A. R. A., Gerlee P. (2013). A mathematical model of tumour selfseeding reveals secondary metastatic deposits as drivers of primary tumour growth. *Journal of the Royal Society Interface*.

[20] Espinosa L. A., Jamadar D. A., Jacobson J. A. (2008). CT-guided biopsy of bone: A radiologist's perspective. *American Journal of Roentgenology*.

