# Supplementary figures and images for: Evaluation of Tumor Viability for Primary and Bone Metastases in Metastatic Castration-Resistant Prostate Cancer Using Whole-Body Magnetic Resonance Imaging
Source: Case Rep Urol. 2018 Jun 7;2018:4074378. doi: 10.1155/2018/4074378 (PMC6011115; doi:10.1155/2018/4074378)

Supplementary Materials


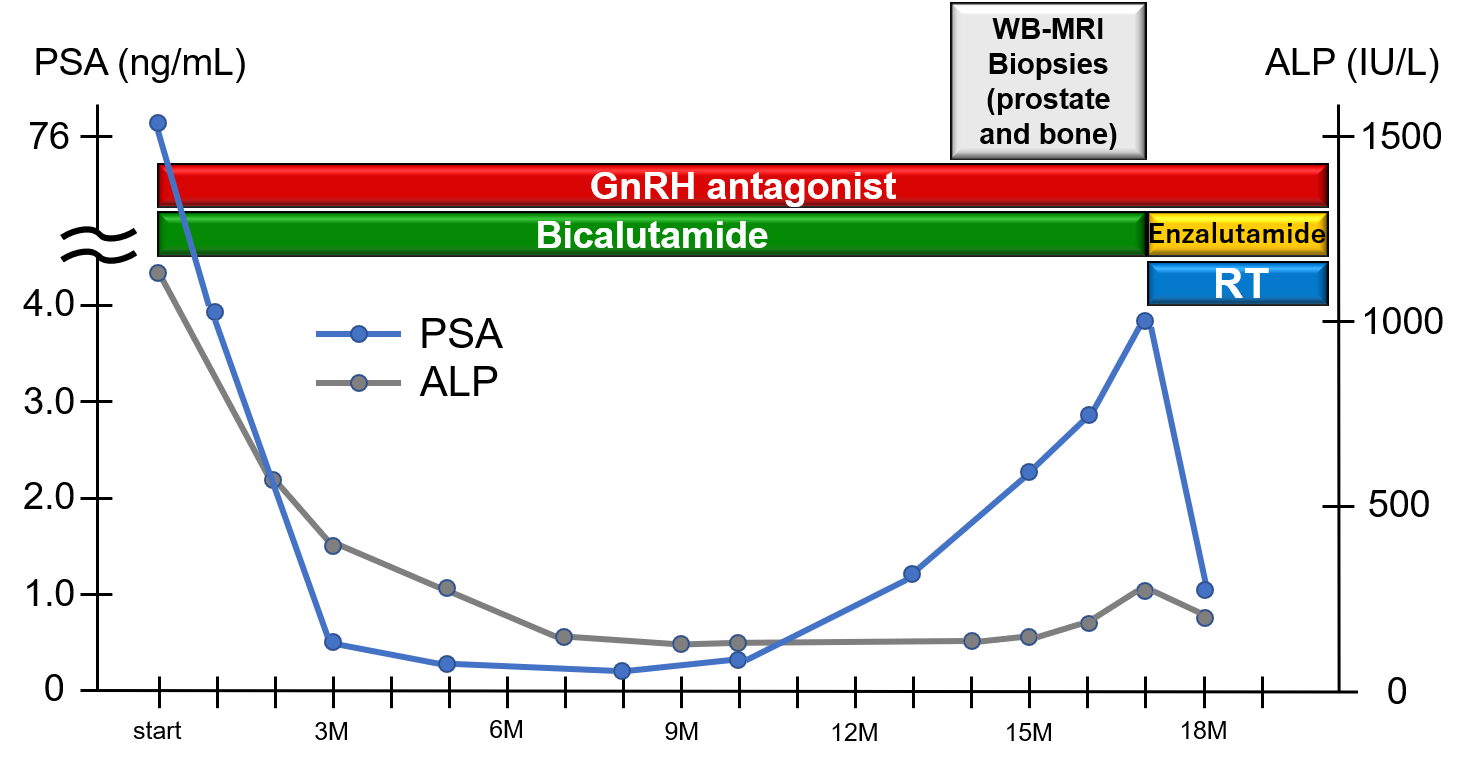

Supplement: Supplementary Materials — The course of treatment of the patient is demonstrated. His elevated PSA immediately declined and was controlled at a level of <0.2 ng/mL after the prostate RT and the administration of enzalutamide. [file 4074378.f1.docx]
